# Call to action regarding the vascular‐bipolar link: A report from the Vascular Task Force of the International Society for Bipolar Disorders

**DOI:** 10.1111/bdi.12921

**Published:** 2020-06-08

**Authors:** Benjamin I. Goldstein, Bernhard T. Baune, David J. Bond, Pao‐Huan Chen, Lisa Eyler, Andrea Fagiolini, Fabiano Gomes, Tomas Hajek, Jessica Hatch, Susan L. McElroy, Roger S. McIntyre, Miguel Prieto, Louisa G. Sylvia, Shang‐Ying Tsai, Andrew Kcomt, Jess G. Fiedorowicz

**Affiliations:** ^1^ Centre for Youth Bipolar Disorder Sunnybrook Health Sciences Centre Toronto ON Canada; ^2^ Departments of Psychiatry & Pharmacology Faculty of Medicine University of Toronto Toronto ON Canada; ^3^ Department of Psychiatry and Psychotherapy University of Münster Münster Germany; ^4^ Department of Psychiatry Melbourne Medical School The University of Melbourne Melbourne VIC Australia; ^5^ The Florey Institute of Neuroscience and Mental Health The University of Melbourne Parkville VIC Australia; ^6^ Department of Psychiatry and Behavioral Science University of Minnesota Medical School Minneapolis MN USA; ^7^ Department of Psychiatry Taipei Medical University Hospital Taipei Taiwan; ^8^ Department of Psychiatry School of Medicine College of Medicine Taipei Medical University Taipei Taiwan; ^9^ Department of Psychiatry University of California San Diego San Diego CA USA; ^10^ Department of Psychiatry University of Siena Siena Italy; ^11^ Department of Psychiatry Queen’s University School of Medicine Kingston ON Canada; ^12^ Department of Psychiatry Dalhousie University Halifax NS Canada; ^13^ National Institute of Mental Health Klecany Czech Republic; ^14^ Department of Psychiatry and Behavioral Neuroscience University of Cincinnati College of Medicine Cincinnati OH USA; ^15^ Lindner Center of HOPE Mason OH USA; ^16^ Mood Disorders Psychopharmacology Unit University Health Network Toronto ON Canada; ^17^ Department of Psychiatry Faculty of Medicine Universidad de los Andes Santiago Chile; ^18^ Mental Health Service Clínica Universidad de los Andes Santiago Chile; ^19^ Department of Psychiatry and Psychology Mayo Clinic College of Medicine and Science Rochester MN USA; ^20^ Department of Psychiatry Massachusetts General Hospital Boston MA USA; ^21^ Department of Psychiatry Harvard Medical School Cambridge MA USA; ^22^ Hope+Me—Mood Disorders Association of Ontario Toronto ON Canada; ^23^ Departments of Psychiatry, Internal Medicine, & Epidemiology Carver College of Medicine University of Iowa Iowa City IA USA

**Keywords:** atherosclerosis, bipolar disorder, cardiovascular disease, prevention, stroke, vascular

## Abstract

**Objectives:**

The association of bipolar disorder with early and excessive cardiovascular disease was identified over a century ago. Nonetheless, the vascular‐bipolar link remains underrecognized, particularly with regard to how this link can contribute to our understanding of pathogenesis and treatment.

**Methods:**

An international group of experts completed a selective review of the literature, distilling core themes, identifying limitations and gaps in the literature, and highlighting future directions to bridge these gaps.

**Results:**

The association between bipolar disorder and vascular disease is large in magnitude, consistent across studies, and independent of confounding variables where assessed. The vascular‐bipolar link is multifactorial and is difficult to study given the latency between the onset of bipolar disorder, often in adolescence or early adulthood, and subsequent vascular disease, which usually occurs decades later. As a result, studies have often focused on risk factors for vascular disease or intermediate phenotypes, such as structural and functional vascular imaging measures. There is interest in identifying the most relevant mediators of this relationship, including lifestyle (eg, smoking, diet, exercise), medications, and systemic biological mediators (eg, inflammation). Nonetheless, there is a paucity of treatment studies that deliberately engage these mediators, and thus far no treatment studies have focused on engaging vascular imaging targets.

**Conclusions:**

Further research focused on the vascular‐bipolar link holds promise for gleaning insights regarding the underlying causes of bipolar disorder, identifying novel treatment approaches, and mitigating disparities in cardiovascular outcomes for people with bipolar disorder.

## 1 INTRODUCTION

Vascular disease is an exceedingly common, yet arguably underrecognized source of morbidity and mortality in bipolar disorder. The association between bipolar disorder and vascular disease is large in magnitude, consistent across studies internationally, and independent of confounding variables where assessed. The vascular‐bipolar link is multifactorial and is difficult to study given the latency between the onset of bipolar disorder, often in adolescence or early adulthood, and subsequent vascular disease, which usually occurs decades later. As a result, studies have often focused on risk factors for vascular disease or intermediate phenotypes, such as structural and functional vascular imaging measures. While there are multiple relevant mediators of this relationship, including lifestyle (eg, smoking, diet, and exercise), medications, and systemic biological mediators (eg, inflammation), there are few treatment studies that deliberately engage these mediators, and thus far no treatment studies have focused on engaging vascular imaging targets. It is acknowledged that bipolar disorder is not unique with regard to its association with vascular disorder. However, the magnitude of increased risk—and the extent of prematurity—of vascular disorders exceeds what is observed in most other psychiatric conditions. Moreover, vascular risk appears to travel in family pedigrees of adolescents and adults with bipolar disorder, invoking potential genetic contributors to this link.

Experts from the International Society of Bipolar Disorders Vascular Task Force selectively reviewed the literature to synthesize key findings across several domains relevant to the link between bipolar disorder and vascular disease. In addition, the perspective of stakeholders with lived experience of bipolar disorder was represented by a consumer advocacy leader (AK). This paper addresses disparities in treatment of cardiovascular risk factors (CVRFs) and cardiovascular disease (CVD) among those with bipolar disorder, as well as aspects relevant to special populations. We endeavor to provide a broad overview of multiple domains, and future detailed reviews on subtopics herein are anticipated.

## 2 NATIONWIDE STUDIES REGARDING CARDIOVASCULAR MORTALITY AND CARDIOVASCULAR DISEASE IN BIPOLAR DISORDER

Bipolar disorder is consistently associated with an elevated risk of cardiovascular mortality. In contrast to the elevated risk of suicide mortality, which is widely recognized and comprises a core focus of prevention and treatment strategies, excess cardiovascular mortality among people with bipolar disorder is not widely appreciated and is not a core focus of prevention and treatment. Excessive risk of cardiovascular mortality has arguably been best demonstrated in Scandinavian countries with registry data that can be linked to mortality statistics, allowing a population‐based assessment of risk. Osby et al assessed mortality in a cohort of 15 386 inpatients with bipolar disorder from 1973 to 1995.^1^ Bipolar disorder conferred a substantially increased risk of mortality with a Standardized Mortality Ratio (SMR) of 2.6 with 33% of the excess deaths being due to vascular disease (cardiovascular and cerebrovascular) for which the SMR was 2.2.^1^ An elevated risk of cardiovascular mortality was also seen in a Danish registry‐based study of 11 648 first admissions for bipolar disorder (SMR of 1.6).^2^ A pooled estimate for the SMR for cardiovascular deaths in bipolar disorder for studies published between 1987 and 2007 was 1.9.^3^ Cardiovascular SMR may differ according to age; for example, the cardiovascular SMR in a recent population‐based cohort study in Sweden was 8 for adults <40 years old and 2.5‐4 for adults ≥40 years old.^4^ Key mortality studies from 2008 to present are highlighted in Table 1.^4^, ^5^, ^6^, ^7^, ^8^, ^9^, ^10^, ^11^, ^12^

**TABLE 1:** Key studies on cardiovascular mortality in bipolar disorder from past decade

Author year	Sample	Key findings
Almeida et al 2014^11^	Representative Australian cohort of 37 892 men (N = only 101 with bipolar disorder).	23 of 101 men with bipolar disorder died of CVD (SHR 1.4, 95% CI 0.8‐2.1)
Angst et al 2013^12^	Prospective cohort of 403 patients with mood disorder	SMR for CVD of 1.60 for bipolar II disorder, 1.99 for bipolar I disorder, and 3.17 for mania (without major depression)
Boden et al 2015^10^	Population‐based Swedish cohort after first diagnosis of myocardial infarction (SWEDEHEART n = 209 592, bipolar disorder n = 442)	63% higher overall mortality with bipolar disorder (and schizophrenia) after myocardial infarction, even after adjusting for age, gender, smoking, diabetes, hypertension, heart failure, stroke, peripheral vascular disease, infarction type, Killip classification and biomarker levels, and treatment (acute and preventative)
Castagnni et al 2013^9^	Danish Psychiatric Register (n = 3200 with bipolar disorder) after mean follow‐up of 6.6 years.	SMR for CVD of 2.1 (95% CI 1.3‐3.5) in bipolar disorder, but based on 15 CVD deaths
Crump et al 2013^6^	Sample of Swedish inpatients and outpatients of 6 587 036 (n = 6 618 with bipolar disorder).	aHR for CVD of 2.14 (95% CI 1.88‐2.45) in women (219 CVD deaths) and 1.73 (95% CI 1.48‐2.02) in men (162 CVD deaths) after adjusting for age, marital status, education, employment, income, and substance use disorders
Hayes et al 2017^5^	Representative UK cohort identified from primary care electronic medical records from 2000 to 2014 (N = 17 314 with bipolar disorder and 219 387 controls matched by age and gender)	Widening overall mortality gap for bipolar disorder over the time period sampled. Total of 59 CVD deaths observed in those with bipolar disorder; health‐ and behavior‐adjusted HR 1.05 (non‐significant) for CVD death in bipolar disorder
Laursen et al 2013^8^	Register‐based study from Denmark, Finland, and Sweden (n = 39 375 with bipolar disorder).	SMR for CVD mortality in bipolar disorder range 1.6‐2.0 across Nordic countries and gender, life expectancy 11‐20 years less
Laursen et al 2014^7^	Danish registry with 1 061 532 persons (14 317 person years of bipolar disorder follow‐up)	Those with bipolar disorder less likely to be treated with cardiovascular medications. In people without prior myocardial infarction or cerebrovascular disease, those with treatment for CVD risk factors had mortality rate ratio of 2.59 (95% CI 1.43‐4.69) and those not treated had a mortality rate ratio of 3.39 (95% CI 1.87‐6.15)
Westman et al 2013^4^	Population‐based sample of Sweden (n = 17 101 with bipolar disorder) with 20 year follow‐up	Mortality rate ratio of 2.03 (95% CI 1.93‐2.13) for CVD in bipolar disorder.

Abbreviations: CVD, cardiovascular disease; UK, United Kingdom.

The highlighted study by Crump et al included both inpatients and outpatients in Sweden, although likely still underascertained bipolar disorder, with 6618 identified cases from a national cohort of 6.5 million.^6^ Those with bipolar disorder died 9 years younger than expected and this elevated risk of premature (all cause) mortality persisted after adjusting for age, sociodemographic variables, and substance use. The fully adjusted estimates showed significant associations with death from CVD (HR 2.1, 95% CI 1.9‐2.5, for women and 1.7, 95% CI 1.5‐2.0, for men). Similar magnitude estimates were seen for mortality from ischemic heart disease and stroke. Interestingly, the risk factors for CVD were not as strongly associated in this sample (HR of 1.7 in women and 1.6 in men for diabetes mellitus, and there was no increase in the risk of hypertension or lipid disorders). When stratified by prior diagnosis of chronic diseases, such as heart disease and diabetes, the hazard ratio for mortality from these diseases associated with bipolar disorder was significantly lower, leading the authors to conclude “better provision of primary care may effectively reduce premature mortality.^6^” It has indeed been found that those with bipolar disorder appear undertreated for these CVRFs and that such undertreatment is associated with greater mortality risk.^7^

Despite increasing recognition of this disparity, the most recent studies demonstrate that the mortality gap appears to be only widening, even in those nations with the most equitable access to healthcare. A follow‐up study by Osby et al in Sweden found that the excess mortality, due to CVD, increased over the period of 1987‐2010, most significantly among young adults.^13^ A study based in the UK also found that the mortality gap for bipolar disorder rose over the period from 2000 to 2014, with a HR for cardiovascular mortality of 1.9 for the period 2010‐2014.^5^

The aforementioned studies were all based on cases of bipolar disorder identified based on health service utilization. Representative population studies, that do not rely on or require treatment seeking, circumvent concerns regarding biased sampling. Such population‐based studies also show evidence of excess incidence and premature onset of CVD. For example, a longitudinal study evaluating a 3‐year period in a US representative sample (NESARC) showed an elevated incidence of CVD (self‐reported physician diagnosis) in individuals with bipolar disorder compared to those with major depressive disorder and controls. After controlling for confounding factors, adults with bipolar I (n = 1047) and bipolar II (n = 392) disorder were more than twice as likely to report a CVD diagnosis. Onset of CVD occurred at much younger ages among people with bipolar disorder than people without mood disorders (17 years younger for bipolar I disorder, and 14 years younger for bipolar II disorder) and significantly younger even than people with major depressive disorder (n = 4396).^14^ Of note, only one‐quarter of adults with bipolar disorder in this study reported lifetime exposure to antimanic treatment, and such exposure was not associated with increased risk of CVD.

### 2.1 Future directions

There is consistent and compelling evidence linking bipolar disorder to excess cardiovascular mortality, with the strongest evidence coming from northern European patient registry samples. There is also evidence of increased and premature CVD in representative population samples. There remains a need for mortality studies in bipolar disorder with a more global representation, as well as a need to examine mortality in representative population samples (ie, not solely samples based on health service utilization). Insights generated from such studies would be bolstered by inclusion of a broad spectrum of biological, environmental, and traditional CVRFs.

## 3 CLINICAL COHORT STUDIES OF CARDIOVASCULAR MORTALITY IN BIPOLAR DISORDER

The above‐described nationwide studies have high power to detect differences in cardiovascular mortality, but lack the detailed characterization of bipolar samples possible in clinical cohort studies. One of the earliest studies that reported on CVD mortality in a clinical sample of patients with bipolar disorder was by Tsuang et al (1980). In a retrospective cohort study of 100 patients admitted for mania in the Iowa Psychiatric Hospital, with up to 40 years of follow‐up, there was an increased risk of mortality due to diseases of the circulatory system among women.^15^ A report from the International Group for the Study of Lithium‐treated patients (IGSLi) using a longitudinal follow‐up for an average of 6.8 years, which included 727 patients with unipolar or bipolar disorder treated with lithium, showed that there is an increased mortality risk due to cardiovascular causes in these patients compared with the general population [SMR 7.69 (95% CI 1.58, 22.48)], which appeared to be ameliorated with lithium treatment.^16^

The findings of two convergent studies suggest that greater mood symptom burden confers increased risk for cardiovascular mortality. A longitudinal study that compared cardiovascular mortality in 288 bipolar I and 147 bipolar II patients, with an average follow‐up of 16.3 years, found that the manic/hypomanic symptom burden was related to the excess mortality independently from other risk factors (hazard ratio [HR] 1.48, 95% CI 1.16, 1.89, *P* = .002), highlighting a potentially more deleterious effect of manic states in the course of CVD. No medication classes were associated with increased risk, and selective serotonin reuptake inhibitors were associated with decreased risk, of cardiovascular mortality.^17^ The association of affective symptom burden with cardiovascular mortality was further assessed in a sample of 1716 patients with bipolar I disorder. The duration of the most severe depressive episode—but not of the most severe manic episode—was related to increased vascular mortality (HR 1.16, 95% CI 1.02, 1.33).^18^ These findings were in contrast with a previous longitudinal study that included patients with bipolar, schizoaffective, and major depressive disorder, which did not find an association of the persistence of depressive symptoms and subsequent cardiovascular morbidity over a mean time period of 11 years.^19^

Finally, another longitudinal population‐based study comprising 334 bipolar I patients and 334 age‐ and sex‐matched controls from Minnesota (US) assessed the incident risk of fatal and non‐fatal myocardial infarction and stroke in a 30‐year period. When myocardial infarction and stroke were considered as a composite outcome, there was a significant risk in the unadjusted analysis but it was not retained after controlling for baseline CVRFs.^20^ Given the inclusion of multiple covariates despite the small sample size, methodologic limitations render this study inconclusive.

### 3.1 Future directions

There is great need for long‐term longitudinal studies of well‐phenotyped prospective samples to better understand how the varied course of illness and treatments received by patients may influence the risk of CVD and related comorbidities. Due to the lag between exposure and outcome, incorporation of putative mediators and intermediate vascular phenotypes/ biomarkers into these study designs would be valuable.

## 4 TRADITIONAL CARDIOVASCULAR RISK FACTORS

Clinical and epidemiologic studies from multiple countries demonstrate that there is excess prevalence of traditional CVRFs among people with bipolar disorder. Indeed, CVD risk may even be elevated among unaffected first‐degree relatives of persons with bipolar disorder.^21^, ^22^ Recent meta‐analyses confirm increased prevalence and/or incidence rates of obesity,^23^ hypertension,^24^ diabetes,^25^ and cigarette smoking^26^ among individuals with bipolar disorder. Summary estimates from these meta‐analyses for the associations of bipolar disorder with obesity, hypertension, and diabetes are reported in Table 2 and show stronger associations with type 2 diabetes and obesity than hypertension. The burden of CVRFs may differ internationally, with greater burden of CVRFs in North America as compared to Europe.^25^, ^27^ Although increased CVRFs are not unique to bipolar disorder, there is evidence that the prevalence of CVRFs among people with BD is higher not only in comparison to controls but also in comparison to people with major depressive disorder.^28^

**TABLE 2:** Summary results from meta‐analysis on the associations between cardiovascular risk factors and bipolar disorder

Author	Risk factor	Sample	Summary finding
Alyerbe et al 2018^24^	Hypertension	Three population‐based studies (Denmark, Taiwan, UK) together comprising more than 1.4 million people.	IRR 1.27 (95% CI 1.15‐1.40)
Vancampfort et al 2016^25^	Type 2 Diabetes Mellitus	Six studies of varied designs and unspecified locations with a combined N = 4688	RR 1.89 (95% CI 1.29‐2.77)
Zhao et al 2016^23^	Obesity	Nine cross‐sectional studies spanning several North American, South American, and European countries as well as Korea; N = 12 259 with bipolar disorder and N = 615 490 controls	OR 1.77 (95% CI 1.40‐2.23)

Abbreviations: CI, confidence interval; IRR, incidence rate ratio; OR, odds ratio; RR, relative risk; UK, United Kingdom.

In addition to concerns from a physical health perspective, the presence of CVRFs among those with bipolar disorder signals an elevated risk for more severe psychiatric symptoms. For example, obesity in bipolar disorder is associated with greater mood symptom burden.^27^, ^29^, ^30^ Remarkably, obesity in bipolar disorder is associated with increased rates of suicide attempts in clinical and epidemiologic studies internationally, in both adults and youth.^27^, ^31^, ^32^, ^33^, ^34^, ^35^, ^36^ Although most findings are retrospective, prospective studies confirm that obesity predicts increased risk for mood episodes,^37^, ^38^ and reduced response to treatment (both pharmacologic and psychosocial) of manic and depressive symptoms.^39^, ^40^, ^41^ Although less widely studied, similar findings are observed for other CVRFs including diabetes,^42^, ^43^ hypertension,^44^ and smoking.^45^, ^46^, ^47^, ^48^

CVRFs are also associated with reduced neurocognitive function, particularly in frontal‐executive tasks.^49^, ^50^, ^51^, ^52^, ^53^, ^54^, ^55^, ^56^, ^57^ The strength of this association may depend in part on the symptomatic status and on the illness duration/stage.^58^, ^59^ Prospective studies indicate that the association among CVRFs, particularly obesity, and impaired cognition is bidirectional.^53^, ^59^ The association of smoking with neurocognition is inconsistent, possibly because nicotine has dopaminergic properties that may counteract any vascular‐related neurocognitive risk.^60^, ^61^, ^62^

Finally, CVRFs are also associated with overall functioning. Many,^42^, ^52^, ^63^, ^64^, ^65^ although not all,^66^, ^67^ cross‐sectional studies have found that among individuals with bipolar disorder those with, vs without, CVRFs such as obesity, metabolic syndrome, and diabetes had poorer psychosocial functioning and higher rates of disability. Obesity^67^ and clinically significant weight gain^66^ may predict less improvement in functioning following mood episodes, whereas weight loss may yield improved functioning.^68^

### 4.1 Future directions

While bipolar disorder is strongly related to a myriad of CVRFs, greater prospective research to discern temporality and mediators is needed. A better understanding of the bidirectional influence of these vascular comorbidities on bipolar disorder could help us understand how they may moderate outcomes and impact treatments. Whereas there are many cohort studies in bipolar disorder that carefully characterize the prospective symptomatic course using repeated measures, there is a paucity of studies that integrate similar repeated measures of CVRFs. Such studies are greatly needed, and would benefit from including measures of lifestyle (see Section 5) and biological (see Section 7) mediators.

## 5 LIFESTYLE MEDIATORS

Many behaviors associated with bipolar disorder may serve as mediators of risk for vascular disease among individuals with bipolar disorder. These are important to recognize as they may be potentially modifiable and targets for intervention.

Smoking is 2 to 3.5 times more common among persons with bipolar disorder than the general population, with smoking prevalence estimates ranging from 30% to 70%.^26^, ^69^ Indeed, smoking may be a risk factor for developing bipolar disorder.^70^ Smoking cessation treatment, which may include counseling with or without adjunctive medication, has not been studied in randomized trials in samples with bipolar disorder alone. However, a large randomized controlled trial in which 70% of participants had a mood disorder found that varenicline was more effective than nicotine replacement or bupropion and all active treatments were more effective than placebo.^71^ Adverse neuropsychiatric events were infrequent and not more common with active treatment relative to placebo.^71^ Preliminary findings from a study of 65 outpatients with schizophrenia, schizoaffective disorder, or bipolar disorder found that varenicline improves Framingham estimates of cardiovascular risk, despite the fact that relapse to smoking is common as is substantial weight gain among those with sustained remission.^72^

Sleep disturbances are a risk factor for CVRFs such as obesity and hypertension, and are also common in individuals with bipolar disorder in all phases of illness including remission. In one study, 55% of an interepisode sample with bipolar disorder met diagnostic criteria for insomnia.^73^ Sleep disturbances are associated with weight gain,^74^ and among individuals with bipolar disorder, with more severe mood symptomatology and poorer treatment response.^75^ Thus, addressing sleep disturbances could be an adjunctive intervention to reduce cardiovascular risk, although this has not been studied. For patients with sleep disturbance, pharmacotherapy (ie, melatonin or melatonin agonists)^76^, ^77^ and psychosocial interventions can be helpful. For example, those whose sleep disturbances remain refractory could benefit from interpersonal and social rhythms therapy or a bipolar‐specific modification of cognitive‐behavioral therapy for insomnia (CBTi‐BP), which involves regularizing bedtime and rise times, stimulus control, and cautious use of sleep restriction.

Individuals with bipolar disorder are also more likely to have poor dietary and nutritional habits.^78^, ^79^ Up to 30% of people with bipolar disorder report binge‐eating behavior, which is associated with metabolic risk independent of its effect on obesity.^80^ Moreover, binge‐eating behavior is a predictor for weight gain following treatment with quetiapine.^81^ In addition, several studies suggest that individuals with bipolar disorder are more likely to consume only one meal per day,^79^ a high‐fat and low‐fiber diet,^82^ more saturated fat,^83^ more sucrose and sweetened beverages,^84^ and add more salt to their food.^85^ While the findings relating to nutrition are generally convergent, there are exceptions, such as a study reporting that people with bipolar disorder reported consuming fewer calories, including from carbohydrates and fats, and more fiber, than historical controls—despite having twice the prevalence of metabolic syndrome.^86^ Mood states and mood symptom burden may explain in part these inconsistencies. In addition, evening chronotype may be associated with significantly higher rates of both binge eating and unhealthy eating habits.^87^

The estimated prevalence of a sedentary lifestyle is between 40% and 65% in people with bipolar disorder.^88^ Greater age and greater BMI are associated with particularly low levels of physical activity among individuals with bipolar disorder.^89^ Samples with bipolar disorder also demonstrate a diminished functional exercise capacity, with poor exercise test performance related to depressive symptoms and musculoskeletal complaints.^90^ Greater physical activity is also associated with a reduced burden of depressive symptoms, better quality of life, better overall function, but a greater burden of manic symptomatology.^88^, ^91^ There is reason to believe that exercise may improve mood.^92^, ^93^ In a sample with unipolar depression, exercising 45 min/day for 3 days/week was as effective in reducing depressive symptoms as 50‐200 mg flexibly dosed sertraline at 16 weeks, with a lower risk of recurrence over long‐term (10 month) follow‐up.^94^, ^95^ Non‐randomized pilot data in bipolar disorder are encouraging.^96^ Exercise also appears to increase brain‐derived neurotropic factor (BDNF) levels in women but not men with bipolar disorder,^97^ leading some to hypothesize that exercise promotes neurogenesis.^98^

While there exist a variety of pharmacological and lifestyle interventions seeking to optimize nutrition/diet and/or physical activity in the general population, attempts to study or adapt these interventions for those with bipolar or related disorders have been limited. A pilot study of integrated medical and outpatient psychiatric care for bipolar disorder that included focus on sleep/wake rhythms, nutrition, and exercise improved function and reduced psychiatric hospitalizations; however, this study did not report on the impact on risk factors for CVD.^99^ A larger study targeting a broader sample with serious mental illness (22% bipolar disorder) found that individually tailored weight management with group exercise sessions resulted in a 3.2 kg greater weight loss at 18 months relative to controls.^100^ A lifestyle intervention developed specifically for bipolar disorder, the Nutrition, Exercise, and Wellness intervention (NEW Tx) for bipolar disorder, combines established nutrition/diet and exercise strategies from the general population tailored for those with serious mental illness. Preliminary evidence suggests that the intervention is feasible and acceptable and may encourage exercise in those with bipolar disorder.^101^, ^102^

### 5.1 Future directions

There is a need to directly evaluate smoking cessation interventions in samples with bipolar disorder, which have unique risks such as treatment‐emergent mania. Preliminary evidence of exercise as a treatment for bipolar disorder is a promising area for future research; it is remarkable that there are thus far no published clinical trials that target aerobic/cardiorespiratory fitness in bipolar disorder. Several effective treatments targeting lifestyle have been developed in the general population, but there is a great need to develop treatments tailored for bipolar disorder specifically, rather than “off the shelf” general population approaches that do not adequately integrate such themes as mood symptoms and medication side‐effects. From a methodologic perspective, studies that incorporate objective measures of sleep and physical activity (eg, accelerometry) and that move beyond simple self‐reports of nutrition and diet are needed.

## 6 MEDICATIONS AND THE VASCULAR‐BIPOLAR LINK

### 6.1 Association of psychotropic medications with cardiovascular disease and cardiovascular risk factors

The medications commonly used to treat bipolar disorder adversely affect CVRFs with the potential for substantial changes in weight or other risk factors^103^ (Note: while various psychotropic medications have cardiovascular side‐effects and/or risks such as arrhythmia, hypertensive crisis, and orthostatic hypotension,^104^ the focus of the current article is on atherosclerotic CVD). Widely considered the first‐line mood stabilizer, lithium has been associated with weight gain, but is likely associated with less weight gain than valproate or antipsychotics.^105^ Lamotrigine or carbamazepine do not commonly induce substantive changes in weight.^106^, ^107^ Antipsychotics, particularly second‐generation antipsychotics (SGAs), are known to promote weight gain. Clozapine, olanzapine, iloperidone, chlorpromazine, sertindole, quetiapine, risperidone, and paliperidone appear to confer the greatest risk for weight gain.^103^ The lowest potential for weight gain is seen with ziprasidone, lurasidone, and cariprazine.^103^ Aripiprazole is sometimes also listed as lower in risk, although study results have varied. Aripiprazole is associated with at least double the weight gain observed for adult patients taking mood stabilizers,^108^ and is associated with greater weight gain vs placebo in short‐term studies of youth with bipolar disorder.^109^

Overall, the general propensity of mood stabilizers and antipsychotics to increase the risk of CVRFs underscores the importance of routine monitoring for weight gain and other adverse cardiometabolic side‐effects and appropriate early action where needed. Clearly, treatment‐related CVRFs should be minimized in a population at excessive risk. However, it is important to acknowledge that despite the clearly increased risk of CVRFs from many psychotropic medications, there is not definitive evidence that these medications independently increase the risk of CVD among people with bipolar disorder, and some evidence to refute that hypothesis. The PRIMROSE study found that SGAs are not associated with CVD mortality among people with serious mental illness (including schizophrenia, bipolar disorder, or other non‐organic psychosis).^110^ Even clozapine, perhaps the worst among contemporary psychotropic medications in terms of adversely affecting CVRFs, is associated with reduced mortality, particularly when used consistently.^111^ A recent study of 62 250 Finnish adults with schizophrenia followed for a mean of 14.1 years found that antipsychotics are not associated with cardiovascular hospitalization; moreover, the adjusted hazard ratio for cardiovascular mortality for those taking antipsychotics was 0.62.^112^ How can this be? Improved symptom control may enable better health‐related behaviors and health service utilization, and indirectly impact cardiovascular health. But there may also be more direct vascular‐related effects of antipsychotics. Aside from dopamine‐related properties of antipsychotics, these medications are pleiotropic, and are associated with reduced inflammation, nitric oxide release, calcium homeostasis, and other mechanisms that may mitigate the cardiovascular risk of these medications.^113^, ^114^, ^115^) Such paradoxical associations have also been observed for statins, which reduce cardiovascular risk despite an association with diabetes.^116^ A meta‐analysis of long‐term (≥1 year) trials of antipsychotics did not show any differences in risk for myocardial infarction, stroke, or cardiovascular death.^117^

### 6.2 Potential role of vascular‐related medications as repurposed treatments for bipolar disorder

It has been suggested that the bidirectional associations between CVD and bipolar disorder may represent a pathophysiologic nexus and thus a potential opportunity to repurpose medications traditionally used in the treatment of CVD for bipolar disorder.^118^ Statins, a class of medications that are 3‐hydroxy‐3‐methyl‐glutaryl‐CoA (HmG CoA) reductase inhibitors, have generated interest in terms of preventing and/or treating depression, perhaps by mitigating the negative effects of inflammation and low density lipoprotein cholesterol on mood.^119^, ^120^, ^121^, ^122^, ^123^ A meta‐analysis of 3 small clinical trials (total n = 165) involving augmentation of SSRI treatment of depression with a statin estimated a net benefit (SMD = 0.73) of statin use on depression.^124^ A larger meta‐analysis of statin trials found no effect for overall psychological well‐being (7 trials with total n = 2105), but a sensitivity analysis of the 5 trials analyzing depression showed significant improvements (SMD = 0.43).^125^ However, we are not aware of any studies in bipolar disorder samples. Future treatment studies would need to take cholesterol levels into consideration, given prior evidence that low cholesterol levels may be associated with depression, mania, suicidality, impulsivity, aggression, and mortality.^126^, ^127^, ^128^, ^129^

There has been similar interest in a potential therapeutic role for antihypertensives in the treatment of mood disorders, including bipolar disorder. While some observational studies of calcium channel blockers are encouraging, a meta‐analysis of 6 RCTs of verapamil for mania did not show any significant benefit.^130^ Candidate gene studies suggested a potential role for polymorphisms in angiotensin converting enzyme (ACE) in bipolar disorder.^131^, ^132^ Relative to other antihypertensive treatments, ACE inhibitors were associated with a lower risk for hospital admission for mood disorders from a large hospital database.^133^ A meta‐analysis of 11 trials further suggested a significant, but potentially negligible (SMD ≤0.15), effect of ACE inhibitors and angiotensin receptor antagonists on psychological well‐being,^134^ although these studies were not specifically designed for this purpose.

### 6.3 Future directions

Several medications used to treat bipolar disorder carry a substantial risk for cardiometabolic side‐effects. There is considerable individual variability in vulnerability to these side‐effects; their improved prediction could inform stratification for preventive interventions (eg, lifestyle interventions, metformin, etc). While the extant evidence is not yet strong enough to support the routine use of medications used for the treatment of CVRFs to target psychiatric symptomatology in bipolar disorder, there are some interesting findings related to statins and ACE inhibitors that invite rigorous clinical trials.

## 7 BIOLOGICAL MEDIATORS

There have been numerous peripheral biomarker (ie, derived from blood samples) studies in bipolar disorder, as comprehensively summarized in the ISBD Biomarker Task Force paper.^135^ The most frequently studied peripheral biomarkers in bipolar disorder,^135^, ^136^ namely, inflammatory markers, oxidative stress markers, and BDNF, are also relevant to CVD. Although other systemic biological mediators may also contribute to the vascular‐bipolar link, such as adipokines.^137^, ^138^, ^139^ autonomic dysfunction, and hypothalamic‐pituitary‐adrenal axis hyperactivation,^140^ this section focuses on inflammation, oxidative stress, and BDNF, the putative biological mediators of the vascular‐bipolar link for which there is the greatest evidence to date. Moreover, from a treatment perspective, psychotropic medications generally reduce inflammation and oxidative stress, and increase BDNF in clinical and preclinical studies.^135^, ^141^

### 7.1 Inflammation

Inflammation is associated with incident CVD, traditional CVRFs,^142^ and endothelial dysfunction (ED).^143^, ^144^ Markers of inflammation, most consistently c‐reactive protein (CRP), contribute to the prediction of CVD beyond Framingham risk scores.^142^, ^145^ Putative mechanisms of the association between mood disorders and inflammation include glucocorticoid resistance, blood‐brain barrier disruption, altered neurotransmitter metabolism, impaired functional connectivity, astrocyte and microglia activation, neuronal damage and degeneration, and reduced neurotrophic support.^146^ In adult bipolar disorder, increased frontal cortical inflammation has been observed in postmortem samples^147^ and pro‐inflammatory genotypes have been implicated.^148^ Numerous studies on the topic, including several meta‐analyses, confirm that peripheral pro‐inflammatory markers are elevated in bipolar disorder, particularly during symptomatic intervals of mania and depression.^149^, ^150^

### 7.2 Oxidative stress

Oxidative stress, reflecting an imbalance between oxidants and antioxidants, is relevant to vascular function, atherosclerosis, and CVD.^151^, ^152^ The interaction of reactive oxygen species with endothelium‐derived nitric oxide contributes to ED which is in turn a precursor to atherosclerosis.^153^ Oxidative stress‐related endothelial damage contributes both to atherosclerotic disease and to metabolic conditions that confer risk for CVD, such as diabetes, dyslipidemia, and hypertension.^154^ These effects are, in part, related to shared genetic susceptibility to oxidative stress and CVD.^155^ As is common in articles regarding novel therapeutic approaches in bipolar disorder, the concept of therapeutically targeting mitochondrial function for the purpose of reducing oxidative stress is also relevant to CVD.^156^ Postmortem studies in bipolar disorder have shown elevated oxidative stress in the brain, especially in frontal regions.^157^, ^158^ Similar to the inflammation literature, studies consistently demonstrate elevations of peripheral oxidative stress markers in bipolar disorder, particularly during symptomatic intervals. Oxidative stress has been posited to contribute to neuroprogression.

### 7.3 BDNF

Whereas the links between CVD and inflammation/oxidative stress are more widely recognized, and perhaps more self‐evident, there is also a substantial literature regarding the implications of BDNF.^159^ Reduced BDNF levels have been observed in adults suffering from acute coronary syndrome.^160^ Serum BDNF levels were inversely associated with CVD and CVD mortality in a prospective study of 3687 Framingham participants, and BDNF genotypic analysis in a related study suggests that the protective effect may be causal.^161^ Even in young adults, BDNF levels are associated with vascular function and functional capacity for exercise.^162^ Deficiency of BDNF may be implicated in reduced endothelial integrity and increased endothelial cell apoptosis.^163^ The BDNF valine to methionine substitution at codon 66 (val66met) polymorphism, associated with reduced function, is associated with bipolar disorder in children and adolescents, and with early‐onset bipolar disorder in adults, and may contribute to prefrontal cortical morphometric and metabolic abnormalities in bipolar disorder.^164^, ^165^ A systematic review and meta‐regression analysis (n = 548 bipolar disorder, n = 565 controls) reported reduced BDNF with large effect sizes (ES) for mania (ES –0.81) and depression (ES –0.97) vs controls.^166^ In contrast, differences in BDNF levels among euthymic bipolar disorder patients vs controls were not significant and of modest magnitude (ES –0.20).

### 7.4 Future directions

While several putative biomarkers have been identified, little is known about the temporal nature of these relationships and/or whether they are causal. Prospective study would be important to discern the relation between these biomarkers and the course of BD and atherosclerosis/CVD. Finally, target‐engagement clinical trials are warranted to evaluate whether modifying these biomarkers has an influence on the development and/or progression of CVD.

## 8 VASCULAR‐RELATED NEUROIMAGING

### 8.1 Leukoaraiosis/white matter hyperintensities

Hyperintensities in brain MRI are thought to reflect focal ischemic damage. Increased rates of white matter hyperintensities (WMH), also described as leukoaraiosis, is among the most consistent structural neuroimaging findings in bipolar disorder.^167^, ^168^ While described as non‐specific with regard to underlying cause, leukoaraiosis is often considered a manifestation of cerebral small vessel disease, and is an independent predictor of stroke and dementia.^169^ Meta‐analysis of 27 studies found significantly increased rates of deep WMH among those with bipolar disorder compared to controls (OR 3.2, 95% CI 2.2, 4.5).^170^ Rates of overall MRI hyperintensities were numerically but non‐significantly higher in bipolar disorder than in major depressive disorder (OR 1.6, 95% CI 0.9‐2.7) and schizophrenia (OR 1.5, 95% CI 0.9, 2.7). The risk of hyperintensities relative to controls was greatest in youth (OR 5.7, 95% CI 2.3, 13.7). There is evidence that WMH burden is related to familiality and bipolar disorder subtypes,^171^ although findings in relatives of people with bipolar disorder are inconsistent.^172^ Despite questions regarding whether the association between bipolar disorder and WMH is explained in part by comorbidities such as migraine, developmental disorders, and/or CVRFs,^173^ the consistency of increased WMH in bipolar disorder alongside the association of WMH with systemic and cerebral vascular disease indicates the need for continued research on this topic.

### 8.2 Neuroimaging correlates of cardiovascular risk factors

Obesity among otherwise healthy people is associated with significantly lower total brain volume, particularly lower grey matter volume,^174^, ^175^, ^176^, ^177^, ^178^, ^179^ and with greater brain volume loss over time.^180^ These findings have led a number of research groups to investigate whether obesity is associated with brain changes in bipolar disorder. In a first‐episode mania sample, overweight/obese bipolar disorder patients had lower white matter volumes than normal‐weight patients, particularly in the frontal and temporal lobes and subcortical white matter.^181^, ^182^ ^)^ A second study, also in first‐episode mania patients, reported lower temporal‐parietal‐occipital white matter integrity in overweight/obese compared to normal‐weight patients.^183^ BMI‐related gray matter reductions have also been detected early in the course of bipolar disorder. In an adolescent bipolar disorder sample, higher BMI was associated with lower orbitofrontal cortical and prefrontal cortical thickness.^184^ Brain chemistry has been less well studied. In one first‐episode mania sample, higher BMI predicted greater hippocampal glutamate and lower hippocampal N‐acetylaspartate (NAA) in bipolar disorder patients.^185^, ^186^ ^)^ Interestingly, this was independent of any effect of BMI on hippocampal volumes, since two separate groups reported that hippocampal volume did not vary with BMI in bipolar disorder patients.^186^, ^187^ That BMI‐related brain changes were located primarily in limbic brain areas, and that they were detected in bipolar disorder patients but not age‐ and gender‐matched control subjects, suggests that higher BMI is associated with unique brain changes in bipolar disorder, such that frontal/temporal limbic brain changes characteristic of bipolar disorder are more pronounced with higher BMI. In keeping with this hypothesis, the only prospective study to date found that first‐episode mania patients with clinically significant weight gain (≥7% of baseline weight) over 12 months also had significantly greater volume loss in the left anterior cingulate gyrus, orbitofrontal cortex, and middle temporal gyrus over the same time period.^188^ However, the observational nature of the study precludes definitive conclusions regarding the direction of the observed associations. Moreover, “third variable” causes such as genes that impact both BMI and limbic brain structure/function cannot be excluded.^189^, ^190^, ^191^

Comparatively fewer studies have examined dysglycemia (ie, diabetes or insulin resistance/glucose intolerance) in relation to neuroimaging phenotypes in bipolar disorder. Hajek and colleagues reported that dysglycemia among adults with bipolar disorder is associated with lower hippocampal and insular cortex volumes compared to euglycemic adults with bipolar disorder and healthy controls, and is associated with stronger aging effects on hippocampal volumes.^192^ A subsequent MR spectroscopy study from the same group found that dysglycemia among adults with bipolar disorder was associated with reduced prefrontal levels of NAA and total creatine compared to euglycemic adults with bipolar disorder and healthy controls; lower levels of these markers were in turn related to poorer global functioning.^193^ The relationship between cigarette smoking and neuroimaging findings among individuals with bipolar disorder has also received relatively little attention. We are aware of only one study investigating this relationship, which reported that in a mixed sample of bipolar disorder and schizophrenia patients, smokers had reduced cortical thickness in the left anterior cingulate cortex and insula.^194^

### 8.3 Cerebral blood flow

An extensive literature has used neuroimaging to examine the function of large‐scale neural networks in bipolar disorder, identifying consistent patterns of altered neural response to cognitive and emotional tasks and abnormal coordination of brain activity within and between neural circuits.^195^ However, the integrity of the cerebrovascular system, which provides the brain with oxygen and energy substrates, has been comparatively less studied in bipolar disorder. Cerebral blood flow (CBF) is considered an indicator of brain health^196^ that is associated with WMH and with systemic vascular risk factors.^197^, ^198^ Indeed, CBF has been the most common vascular‐related neuroimaging phenotype studied in bipolar disorder. A recent systematic review identified 33 studies with a total of 508 bipolar disorder participants and 538 controls.^199^ CBF measurements included single‐photon emission computerized tomography (SPECT; n = 15 studies), positron emission tomography (PET; n = 8), arterial spin labeling MRI (ASL; n = 7), perfusion weighted imaging (n = 1), and other approaches (n = 2). Most studies of adults with bipolar disorder during episodes of depression or mania found reduced resting CBF in frontal and midline regions vs healthy controls,^200^, ^201^, ^202^, ^203^ but there were exceptions.^204^, ^205^ There was greater inconsistency in findings relating to euthymic bipolar disorder and/or symptomatically heterogeneous groups. Evidence suggests that resting CBF may be most abnormal in those with greater cognitive deficits; Dev et al^206^ found that higher resting CBF was associated with better inhibitory performance in the bipolar disorder group but not in the comparison group. Although most studies have examined CBF at rest, several studies have examined changes in CBF as a function of cognitive and/or emotional tasks. For example, Kruger et al found that, following a sad mood provocation task, CBF in medial frontal cortex decreased significantly in bipolar disorder as compared to healthy controls.^207^ Most studies that evaluated CBF in bipolar vs unipolar depression have not found significant differences, although one study using pattern recognition analysis found that, despite lack of differences from healthy controls, subgenual anterior cingulate cortex CBF classified unipolar vs bipolar depression in females with 81% accuracy.^208^ Finally, with the exception of a single lithium discontinuation study,^209^ there is a paucity of data regarding bipolar disorder treatment and treatment response in relation to CBF and changes therein.

### 8.4 Future directions

Thus far, there is a gap with regard to the link between neuroimaging phenotypes and hypertension/blood pressure or lipids levels,^55^ and related studies are warranted. There are opportunities to capitalize on the advantages of ASL, which magnetically tags blood as a non‐invasive endogenous tracer. This offers advantages in general, but particularly in settings where SPECT or PET are not available, and in populations, such as youth and pregnant women, where ionizing radiation should be avoided. Moreover, use of ASL allows for the examination of CBF in relation to other functional and structural MRI phenotypes within the same imaging session. The concept of using vascular‐related neuroimaging phenotypes as a treatment target, predictor, and/or moderator has received little attention to date; such a concept may afford insights regarding etiopathology and regarding the use of vascular‐related interventions.

## 9 VASCULAR IMAGING AND FUNCTION

The assessment of vascular function and structure provides an opportunity to investigate intermediate phenotypes, prior to the development of overt vascular disease through surrogate outcomes such as ED. This can facilitate an understanding of how mood symptoms, the course of illness, or treatment of illness may influence risk for vascular disease without waiting for the patient to develop cardiovascular events such as myocardial infarction late in life. Moreover, ED that predates frank atherosclerosis can be mitigated using behavioral and pharmacological approaches (see Section 6).

A few cross‐sectional studies have assessed vascular structure and function in adults and adolescents (see Section 10). In adult studies, findings to date have been age dependent. A sample of 27 people with bipolar disorder of a mean age of 32 years did not show any differences compared to matched controls on flow‐mediated dilatation (FMD), nitroglycerin‐mediated dilation of the brachial artery, pulse‐wave velocity (PWV), or augmentation index (AIX).^210^ There were also no differences in the reactive hyperemia index or AIX as measured by finger plethysmography in a larger sample of 56 participants with a mean age of 40 years.^211^ Another group reported albuterol‐induced vasoreactivity deficits among 31 adults (mean age 43 years) with depression; subgroup analyses found significantly lower vasoreactivity among those with unipolar depression (n = 11, *P* = .005) but only nominal lower vasoreactivity among those with bipolar depression (n = 20, *P* = .086).^212^ In another study focused on identifying age‐related effects, the older—but not the younger—half of the sample (median split on 32 years) had greater than expected PWV and AIX relative to normative values and this was related to the retrospectively estimated burden of mood symptoms.^213^ In an older sample with mixed mood disorders, lower FMD was seen in those who had a greater burden of mania/hypomania over more than a quarter century of prior follow‐up in a prospective cohort.^214^

After an initial study regarding retinal vascular photography in adolescents with bipolar disorder,^215^ there have since been several studies on this topic in adults.^216^, ^217^, ^218^, ^219^ One study found that both adults with bipolar disorder and those with schizophrenia had narrower arterioles and wider venules as compared to healthy volunteers, and adults with bipolar disorder had narrower arterioles and wider venues even as compared to adults with schizophrenia,^216^ a pattern generally considered indicative of increased cardiovascular risk.^220^

In summary, non‐invasive measures of vascular function and structure may reflect underlying risk factors for vascular disease. The few small studies on vascular function in adults with bipolar disorder have thus far have only detected differences related to bipolar disorder among older adults, and in proportion to the prior burden of mood symptoms. The link between mood symptom burden and vascular risk has also been supported by studies of cardiovascular mortality in bipolar disorder (see Section 2). Preliminary evidence suggests that retinal vascular calibers that are related to cardiovascular risk are worse among people with bipolar disorder than in healthy volunteers or people with schizophrenia.

### 9.1 Future directions

Non‐invasive measures of vascular structure and function afford the opportunity to study an intermediate phenotype to explore mechanisms without waiting until cardiovascular events or mortality occurs. Thus far, most studies have been based on small sample sizes and cross‐sectional design. In order to move toward implementing these measures clinically, larger, prospective, repeated‐measure studies are needed. Such studies may help identify subgroups for whom these measures are particularly relevant, and would provide a surrogate measure of vascular risk, beyond traditional CVRFs, that may additionally be suited to understanding mechanisms of vascular risk progression overtime and in relation to mood symptoms. In addition to study of medium‐ and large‐caliber vessels, additional studies of microvessels are warranted, such as those involving retinal vascular imaging, and MRI measures of cerebral and cardiac microvessels.

## 10 SPECIAL POPULATIONS: ELDERLY, YOUTH, AND WOMEN/PREGNANCY

### 10.1 Bipolar disorder in youth

Similar to adults, the prevalence of traditional CVRFs, and their clustering, is increased in youth with bipolar disorder.^33^, ^34^, ^221^, ^222^ There is elevated cardiovascular risk among first‐ and second‐degree relatives of youth with bipolar disorder, particularly among relatives of youth with familial bipolar disorder.^223^ As with adults, obesity among youth with bipolar disorder is associated with greater psychiatric complexity, particularly increased suicide attempts in both clinical and largely untreated epidemiologic samples.^33^, ^34^ With regard to CVD‐related lifestyle variables, there is increased cigarette smoking, suboptimal nutrition, and eating behaviors, and reduced physical activity, in comparison to healthy youth.^224^, ^225^, ^226^ Traditional CVRFs are negatively associated with executive function among youth with bipolar disorder, an association not observed among healthy youth.^56^, ^57^ As with adults, there is evidence that inflammation is elevated and BDNF is reduced in youth with bipolar disorder.^227^, ^228^, ^229^ Studies have also found that BDNF, inflammatory markers, and/or oxidative stress markers are associated with traditional CVRFs as well as with non‐invasive ultrasound measures of vascular structure and function.^227^, ^230^, ^231^ Preliminary findings from the first retinal photography study in bipolar disorder suggest retinal vascular caliber is associated with blood pressure, peripheral endothelial function, and mood symptoms in youth with bipolar disorder but not among healthy controls.^215^ In terms of neuroimaging findings, similar to adults, BMI is negatively associated with cortical volumes and structure, particularly in frontal regions.^184^ In contrast to adult studies, there appears to be anomalous elevated CBF in midline and frontal regions among youth with bipolar disorder, although acute aerobic exercise appears to have a temporarily normalizing effect on CBF in youth with bipolar disorder.^184^ Finally, there is evidence of reduced cerebrovascular reactivity, as measured using a standard breath‐hold paradigm during fMRI, among youth with bipolar disorder, and this is especially evident in periventricular and deep white matter, regions where WMH are commonly observed later in life.^232^

### 10.2 Bipolar disorder in the elderly

Patients with bipolar disorder begin to have increased risk of cardiovascular and metabolic morbidities in midlife and may die mainly from CVD before reaching geriatric age, with the average cardiovascular mortality occurring 10 years earlier than the general population.^4^ In light of this premature mortality, patients with early‐onset bipolar disorder who survive into old age probably represent a healthy survivor bipolar disorder subpopulation with favorable outcome. Therefore, studies that focus only on individuals in their 60s and beyond may not be truly representative of the larger bipolar disorder population.^233^ A limited number of studies have focused specifically on bipolar disorder in late life. The prevalence of cardiovascular morbidity including hypertension in older‐age (>60 years) bipolar disorder (OABD) in existing reports appears to be similar to that in community‐based geriatric samples or patients with depressive disorder.^235^, ^236^, ^237^, ^238^ Furthermore, comparable percentages (about 25%) of older patients with bipolar disorder have coronary heart diseases in Asian and Western reports.^238^ However, OABD had significantly higher BMI and a greater burden of endocrine/metabolic diseases.^234^, ^235^, ^237^ Consequently, approximately two‐thirds of OABD are reported to have a previous stroke or silent cerebral infraction (SCI).^239^, ^240^ In particular, SCIs are detected in 59.5% of OABD, which is dramatically higher than the 20% detected in healthy elderly people.^239^ Patients with bipolar disorder, including those with early‐onset illness, are at higher risk of cerebrovascular disease in late life. The number of recurrent affective episodes may increase the risk of stroke in OABD with typical‐onset age.^239^ Cardiovascular morbidity, particularly stroke, may increase with age in OABD and optimal treatment requires awareness of SCI and prevention against recurrent episodes. Among the older (aged >50 years) community‐dwelling patients with serious mental illness, given better social functioning and comparable cognitive function in older bipolar patents, they may remain at higher risk for obesity and medical morbidity than schizophrenic ones. Treatments targeting cognitive impairment and CVDs across their life span are necessary.^241^

### 10.3 Bipolar disorder in women

Emerging evidence suggests that women with bipolar disorder may have a 1.5‐ to 2.5‐fold higher risk for the development of cardiovascular morbidity and for cardiovascular mortality, as compared to women among the general population of Eastern and Western societies.^4^, ^6^, ^242^, ^243^, ^244^ In contrast to the higher risk of CVD in men as compared with premenopausal women in the general population,^245^ there is no gender difference in cardiovascular risk among people with bipolar disorder. Overall, it appears that the extent of increased risk of CVD that is conferred by bipolar disorder is accentuated among women. However, one longitudinal (6‐year) nationwide cohort study with a relatively younger sample (mean age 36 years) showed that females with bipolar disorder had a lower risk of stroke as compared to males.^246^ The difference may result from cardioprotective effects of estrogen.^247^ Nevertheless, women with bipolar disorder are more vulnerable to metabolic syndrome^25^, ^248^, ^249^ along with and CVD gestational hypertension compared to women without psychiatric disorders.^250^, ^251^

### 10.4 Future directions

Thus far, studies in different age groups have been mainly cross‐sectional, whereas longer‐term prospective studies, particularly across key developmental and aging epochs, are needed. Studies of child and adolescent offspring of adults with bipolar disorder are needed, as such study designs are almost uniquely positioned to inform our understanding of the temporal associations between the vascular problems and mood problems to which these offspring are predisposed. Given the known gender differences in the manifestations of, and risk factors for, cardiovascular disorders, future studies should consider gender‐related variables (such as sex hormones, and major shifts therein during developmental and reproductive milestones), and should seek to clarify the mechanisms underlying the apparent excessive vascular burden among women with bipolar disorder.

## 11 DISPARITIES IN CARDIOVASCULAR‐RELATED TREATMENT AND NEED FOR INTEGRATED CARE MODELS

There are fundamental disparities in the screening, preventive intervention, and treatment of CVRFs and CVD among people with serious mental illness, including bipolar disorder.^6^, ^252^, ^253^, ^255^, ^256^ These disparities exist at every conceivable juncture in care (eg, triage, intervention, post‐discharge) or lack thereof. These disparities are also ubiquitous, evident in commercial managed care organizations, US Medicare, the US Veterans’ Affairs, and population‐based data in multiple countries including those with universal healthcare. Finally, these disparities are evident for numerous conditions including diabetes, hypertension, coronary artery disease, congestive heart failure, and stroke. It is worth highlighting a number of specific cardiovascular treatment disparity findings. With regard to hypertension, there appear to be lower rates of screening, prescription, and treatment adherence among people with bipolar disorder.^24^ With regard to diabetes care, there are disparities across multiple benchmarks for people with mental illness; effect sizes are especially large among people with bipolar disorder, and are also proportional to the number of psychiatric comorbidities.^257^ Finally, with regard to cardiovascular mortality, there is compelling evidence of excess cardiovascular mortality alongside undertreatment at multiple levels, even in countries with comprehensive universal healthcare.^254^, ^258^ For example, in Denmark, the increased risk of CVD in bipolar disorder is not reflected in increased treatment contacts for CVD; moreover, even after such contacts are established, there are lower rates of invasive procedures in bipolar disorder. Similar findings are observed in Taiwan, where people with bipolar disorder are half as likely to receive invasive procedures after an acute myocardial infarction.^258^ In case the existence of such disparities was not enough to motivate change, there is compelling evidence that if quality of cardiovascular care post‐myocardial infarction is equivalent, there is no longer excess mortality associated with having a mental illness.^259^ Several studies have sought to evaluate the effectives of integrating physical and mental health within a unified treatment model.^260^, ^261^, ^262^, ^263^ However, such models have not yet been disseminated and/or implemented beyond the original context of a given study, and are therefore not available to most people with bipolar disorder.

### 11.1 Future directions

The aforementioned disparities are multifactorial, and related to a combination of patient factors, provider factors, and system‐level factors. Although preponderance of evidence supports the existence of disparities in care, exceptions exist,^264^ and understanding the mechanisms and factors underlying more equitable service delivery models may yield valuable insights. There is an urgent need for more studies evaluating integrated care settings and pathways, particularly in real‐world clinical and community settings. Finally, a factor not commonly invoked in discussions of treatment disparities is the premature onset of CVD among people with bipolar disorder; simply shifting the frame of cardiovascular focus earlier by a decade would better align prevention and early intervention strategies with the epidemiology of CVD in bipolar disorder. One example of such an approach is that of the American Heart Association, which includes bipolar disorder among conditions that confer increased risk for early CVD, and advises earlier screening for, and tighter control of, CVRFs.^140^ Another example is the PRIMROSE research program, which demonstrated the superiority of modified risk prediction models for people with severe mental illness over models based solely on traditional CVRFs.^110^

## 12 CONCLUSIONS

**FIGURE 1:**
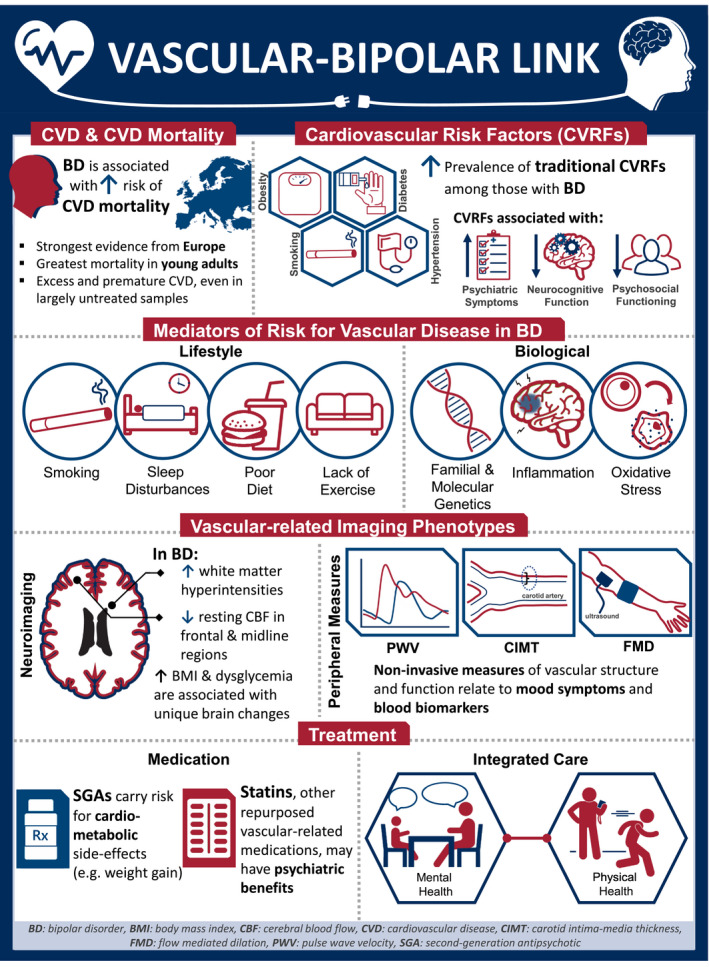
Infographic regarding the vascular‐bipolar link [Colour figure can be viewed at wileyonlinelibrary.com]

The title of this study declares a call to action. The hope of the authors is that this article has served to make explicit the need for such a call (Figure 1), and to illuminate potential future directions. In an era when there has been a marked *reduction* in cardiovascular mortality in the general population, the excess of burden of cardiovascular mortality among people with bipolar disorder has *increased.* We view this discrepancy as outrageous, not because there is a way to address it but because there are numerous ways to address it. But if we focused this call to action solely on the topic of excess CVD and mortality, we would be missing an opportunity to highlight the relevance of blood vessels to the psychiatric and neurocognitive symptoms that contribute to suffering, functional impairment, and reduced quality of life among people with bipolar disorder, beginning at least as early as adolescence. The call to action is aimed at all those for whom treatment, research, education, and/or lived experience of bipolar disorder are important.

Despite the pernicious features of the vascular‐bipolar link, there is also a robust silver lining. How so? Thus far, the treatment of bipolar disorder does not deliberately focus on blood vessels. Given the association of vascular risk factors and disorders with more severe manifestations of bipolar disorder, and given the association of mood symptom burden with vascular disorders and mortality, this raises the question of whether optimizing vascular health can serve to improve mood and cognition. The pipeline of entirely new psychotropic medications has reduced markedly, with little to suggest this will change in the near term. And yet there are evidence‐based pharmacological and behavioral approaches that target vascular health and that have not been adequately studied in bipolar disorder. Relatedly, there is a need for treatment studies that intentionally target vascular phenotypes—including the brain, heart, retinal, and peripheral vessels—for the purpose of evaluating the impact of vascular target engagement on standard bipolar disorder outcomes, and for the purpose of informing personalized treatment approaches. There is a need for prospective cohort studies that integrate vascular measures alongside standard psychiatric measures and measures of putative mediators of the vascular‐bipolar link. Based on the evidence that the vascular‐bipolar link is familial, including relatives in such studies would be an important addition. There is an opportunity for engaging patients and families in a collaborative effort to minimize the drawbacks and maximize the silver linings of the vascular‐bipolar link; knowledge of this link can help those with lived experience advocate for themselves in clinical encounters, and empowers them with the knowledge and hope that optimizing vascular health through lifestyle can benefit both here‐and‐now mental health and longer‐term physical health. Finally, from an antistigma perspective, the vascular‐bipolar link can serve to reduce the separation of bipolar disorder from other complex, chronic medical disorders, and can serve to ensure parity in how bipolar disorder and people with bipolar disorder are understood, prioritized, treated, and researched.

In conclusion, progress regarding the vascular‐bipolar link will require a more deliberate and systematic integration of vascular metrics in research, treatment approaches, healthcare systems, and education of healthcare professionals and the public. We hope that this article will serve to mobilize a broader spectrum of stakeholders with a vested interested in the vascular‐bipolar link, and as such serve as an impetus to accelerate progress. We intend to continue distilling and disseminating the research literature on this topic, including future focused subtopic reviews as the literature and knowledge base continues to accumulate. Finally, we invite any stakeholders who are interested in the vascular‐bipolar link to connect with us in order to share ideas and discuss ways to collaboratively encourage and enable action.

## CONFLICT OF INTEREST

Dr Bond reports honoraria and/or grant funding from Alkermes, Myriad Genetics, the University of Minnesota Foundation, and the University of Minnesota Department of Psychiatry and Behavioral Science. Dr Fiedorowicz has received funding (grants and consultation) from Myriad Genetics, Inc, and grants from National Institute of Mental Health, National Center for Advancing Translational Science and the National Heart, Lung, and Blood Institute. Dr Goldstein reports grant funding from Brain & Behavior Research Foundation, Brain Canada, Canadian Institutes of Health Research, Heart & Stroke Foundation, National Institute of Mental Health, Ontario Mental Health Foundation, Ontario Ministry of Research and Innovation, University of Toronto Department of Psychiatry. Dr Gomes reports speaker honoraria from Abbott, Apsen, Daiichi‐Sankyo, Libbs, and Lundbeck. Dr Hajek reports funding from the Canadian Institutes of Health Research (103703, 106469, and 142255), Brain & Behavior Research Foundation, and the Czech Republic Ministry of Health. Dr McElroy reports having been a consultant to or member of the scientific advisory boards of Allergan, Avanir, Bracket, F. Hoffmann‐La Roche Ltd., Idorsia, Mitsubishi Tanabe Pharma America, Myriad, Opiant, SipNose, Sunovion, and Takeda. She has been a principal or co‐investigator on studies sponsored by Allergan, Avanir, Brainsway, Marriott Foundation, Medibio, Myriad, Neurocrine, Novo Nordisk, Otsuka, and Sunovion. She is also an inventor on United States Patent No. 6 323 236 B2, Use of Sulfamate Derivatives for Treating Impulse Control Disorders, and along with the patent's assignee, University of Cincinnati, Cincinnati, Ohio, has received payments from Johnson & Johnson, which has exclusive rights under the patent. Dr McIntyre reports fees from Takeda, Janssen, Allergan, Otsuka, Shire, Lundbeck, Pfizer, Minerva, Neurocrine, BaushHealth, and NovoNordisk. Dr Prieto has received grant funding from CONICYT of the Government of Chile (grant FONDECYT 1181365 and grant FONDEF ID19I10116). Dr Sylvia reports personal fees from United Biosource Corporation, Clintara, Bracket, and Clinical Trials Network and Institute; royalty fees from New Harbinger; grants from National Institute of Mental Health, Patient Centered Outcomes Research Institute, American Foundation for Suicide Prevention, and Takeda. The other authors do not have any financial disclosures to report.
